# Supplementary material for: Development and validation of clinical prediction models for mortality, functional outcome and cognitive impairment after stroke: a study protocol
Source: BMJ Open. 2017 Aug 18;7(8):e014607. doi: 10.1136/bmjopen-2016-014607 (PMC5724146; doi:10.1136/bmjopen-2016-014607)
Supplement: Author's manuscript [file bmjopen-2016-014607.draft_revisions.pdf]

BMJ Open

DEVELOPMENT AND VALIDATION OF CLINICAL PREDICTION MODELS FOR MORTALITY, FUNCTIONAL OUTCOME AND COGNITIVE IMPAIRMENT AFTER STROKE: A STUDY PROTOCOL

Journal:	*BMJ Open*
Manuscript ID	bmjopen-2016-014607
Article Type:	Protocol
Date Submitted by the Author:	13-Oct-2016
Complete List of Authors:	Fahey, Marion; King's College London, Division of Health and Social Care Research ;<br>Rudd, Anthony; King's College London<br>Béjot, Yannick; Dijon Stroke Registry, EA4184, University Hospital and Medical School of Dijon, neurology<br>Wolfe, Charles; Kings College London, Division of Health and Social Care Research<br>Douiri, Abdel; King's College London, Division of Health and Social Care Research
**Primary Subject Heading**:	Cardiovascular medicine
Secondary Subject Heading:	Epidemiology, Cardiovascular medicine, Patient-centred medicine, Public health
Keywords:	prognosis, Clinical prediction model, Stroke < NEUROLOGY, stratified medicine

SCHOLARONE™
Manuscripts

For peer review only

DEVELOPMENT AND VALIDATION OF CLINICAL PREDICTION MODELS FOR MORTALITY, FUNCTIONAL OUTCOME AND COGNITIVE IMPAIRMENT AFTER STROKE: A STUDY PROTOCOL

Fahey M[1], Rudd AG[1,3], Bejot Y[4], Wolfe C.D.A [1,2], Douiri A[1,2],

Division of Health and Social Care, King's College London, London

[1] Division of Health and Social Care Research King's College London, London, UK

[2]National Institute for Health Research (NIHR) Collaboration for Leadership in Applied Health Research and Care (CLAHRC) South London at King's College Hospital NHS Foundation Trust, UK

[3]National Institute for Health Research (NIHR) Biomedical Research Centre, Guy's and St Thomas' NHS Foundation Trust, London, UK

[4]Registre Dijonnais des AVC, EA4184, Dijon, France.

Corresponding author: Marion Fahey

Corresponding author's address: Division of Health and Social Care Research, Addison House, Guy's Campus, King's College London, London SE1 1UL, UK

Corresponding author's phone and fax: Phone: +44 20 7848 8732;

Corresponding author's e-mail address: marion.fahey@kcl.ac.uk

Word count, excluding title page, abstract, references, figures and tables: 2,468

For peer review only

Abstract

Introduction:Stroke is a leading cause of adult disability and death worldwide. The neurological impairments associated with stroke prevent patients from performing basic daily activities and have enormous impact on families and caregivers. A practical and accurate tool to assist in predicting outcome after stroke at patient level, could provide significant aid for patient management and cost of healthcare delivery. Furthermore, a prediction model of this kind can be useful as a tool for clinical research, health economics, policy making, and clinical decision support.

Methods:The prognostic model will be developed using methods recommended by the Prognosis Research Strategy (PROGRESS) Group and reported using the Transparent Reporting of a multivariable prediction model for Individual Prognosis Or Diagnosis (TRIPOD) statement. 2,869 patients with first ever stroke from South London Stroke Register between 1995-2004 were included in the development cohort. We will construct multilevel models and Cox proportional hazard model to predict recovery trajectories for cognitive impairment, functional outcome and mortality up to 5 years after stroke. Models will be temporally validated using data from 2172 patients recruited to the stroke register between 2005-2014. Additionally, repeated random sub-sampling validation (Monte Carlo cross-validation) will be also evaluated in model development. Data from patients recruited to the Dijon Stroke Register between 1985-2015 will be used for external validation. Discrimination, calibration and clinical utility of the models will be presented.

Ethics:Patients, or for patients with communication problems their relatives, gave written informed consent to participate in stroke-related studies within the SLSR. The SLSR design was approved by the ethics committees of Guy's and St Thomas' NHS Foundation Trust, Kings College Hospital, Queens Square, and Westminster Hospitals (London). The Dijon Stroke Registry was approved by the Comité National des Registres and the InVS and has authorization of the Commission Nationale de l'Informatique et des Libertés.

For peer review only

SUMMARY

Strengths and limitations of this study

- Thorough description of the prognostic model development research pathway, from reviewing literature to external validation.
- First prognostic tool in Stroke to follow, a priori, the Prognosis Research Strategy (PROGRESS) framework, and Transparent Reporting of a multivariable prediction model for Individual Prognosis or Diagnosis (TRIPOD) reporting guidelines for prognostic research.
- Pre specifies statistical analysis plan and informative levels of tool performance to increase transparency of results and of the final report.
- Restricted to only the use of predictor variables measured in previous data sets.
- Minor differences in the way variables are measured between the development and the validation data sets.

For peer review only

INTRODUCTION

Stroke is among the most common cause of serious adult physical disability and the third most common cause of death worldwide (Feigin et al., 2014). Despite the introduction of effective treatments for acute stroke, early rehabilitation and secondary prevention, the majority of stroke survivors have medical comorbidities, physical and/or cognitive impairments that require ongoing active assessment and management (Wolfe et al 2011). Therefore, stroke can be seen as a chronic condition, spanning not only the incident event and formal rehabilitation but the rest of the patient's life. Rehabilitation from stroke requires a sustained, co-ordinated effort from informed multidisciplinary (MDTs) as well as patients and carers, both in the clinical setting and the community (Winstein 2016). MDTs make numerous decisions, often in shared decision making, on the basis of an estimated probability that a specific event will occur in the future in an individual(prognosis). These predictions are used for planning lifestyle or therapeutic decisions on the basis of the risk of developing a particular outcome or state of health within a specific period (Moons et al 2009;Steyerberg et al 2009; Wasson et al 1985). Such estimates can also be used to risk-stratify participants in therapeutic intervention trials (Dorresteijn et al 2011; Hayward et al 2006;Kattan et al 2004). However, information from a single predictor can often be insufficient to provide reliable estimates of prognostic probabilities or risks, particularly in complex patients, for example those with comorbidities (Riley et al 2013;Collins et al 2013). Probability estimates are therefore commonly based on combining information from multiple predictors to form a multivariable clinical prediction model (Roysten et al 2009; Harrell et al 1996).

Clinical prediction models(CPMs), the tests used to identify different patient risk groups, are abundant in prognostic research literature but few are implemented or used in routine clinical practice (Steyerberg, 2013). One explanation for this is that, although models have been developed (Counsell and Dennis 2001; Fahey et al 2016), they have limited utility for clinical applications, particularly in a long term care setting. Progression or regression (recovery) of disease is highly variable both over time and between individuals (Langhorne et al. 2011; Douiri et al 2013). Current clinical prediction models typically estimate risk at predefined time points. With this variability in mind, time series data and methods may be more appropriate for accurately capturing recovery when the aim is planning immediate and long

For peer review only

term care simultaneously for individual patients. There is also generally a lack of confidence among clinicians in applying risk scores in practice. Many believe there is the lack of sufficient evidence to demonstrate the reproducibility and transportability of the model in a different population (Altman 2009). To be considered useful, a risk score should be clinically credible, accurate (well calibrated with good discriminative ability) and have generality (be externally validated) (Altman 2009).

We have evaluated the accuracy of existing models in predicting stroke outcomes by systematic review of literature and meta-analysis. The report concluded no clinical prediction models were sufficiently accurate to become part of routine care (Fahey et al 2016). Models were poorly reported, this further undermines clinicians confidence in the models ability to support patients. One of the recommendations from that research was to build on previous work in developing clinical prediction models suitable for routine care. This aim of this study is the develop and validate (temporally and geographically) clinical prediction models for mortality, functional outcome and cognitive impairment after stroke.

METHODS

Our study design is informed by the PROGRESS framework and specific recommendations for statistical approaches to prognostic research. We will report the study in accordance with the Transparent Reporting of a multivariable prediction model for Individual Prognosis Or Diagnosis (TRIPOD) statement for prediction studies (Collins et al 2015).

The PROGRESS framework outlines four types of prognostic research: (1) fundamental prognosis research; (2) prognostic factor research; (3) prognostic model research; (4) stratified medicine research (Boumester 2012). The proposed study is Type 3, prognostic model research. To develop the proposed clinical prediction models, we will consider the recommendations of Kamper et al and Royston et al (Kamper et al 2011; Royston et al 2009).To validate the prognostic screening tool, we will use the method suggested by Altman et al and Steyerberg et al (Steyerberg et al 2013; Altman et al 2009).

Source of data

The study is a prospective longitudinal cohort study. The data source is the South London Stroke Register(SLSR) an ongoing, prospective population-based stroke register set up in

For peer review only

January 1995 recording all first-ever strokes in patients of all ages for an inner area of South London. The methods of the SLSR have been described previously in detail (Wolfe, 2011; Sarker, 2008) and are summarized here. Stroke was defined according to the World Health Organisation criteria and Oxfordshire Community Stroke Project Classification. Multiple over-lapping sources of information are employed. The key study dates for the derivation and internal validation sample is 1995 to 2004. Temporal validation is from participants from 2004 to 2015.

Participants

Patients admitted to hospitals serving the study area (two teaching hospitals within and three hospitals outside the study area) are identified by regular reviews of acute wards admitting stroke patients, , national data on patients admitted to any hospital in England and Wales with a diagnosis of stroke are screened for additional patients. All general practitioners (N=699 (2011)) within and on the borders of the study area are contacted regularly and asked to notify the SLSR of stroke patients. Referral of non-hospitalised stroke patients to a neurovascular outpatient clinic (from 2003) or domiciliary visit to patients by the study team is also available to general practitioners. Community therapists are contacted every 3 months. Death certificates are checked regularly. Patients are assessed at the stroke onset, 3 months and annually after stroke. Participants receive treatments in line with UK national clinical guidelines for stroke.

Outcome

The start point for these models is time of stroke and outcomes of interest are Mortality (time to event), functional outcome (Bartel Index-) and Cognitive Impairment (MMSE/AMT) up to 5 years after stroke. The outcome definition and measurement method is the same for all patients. Candidate predictors are not part of the outcome. Outcomes type is 'single' i.e. not composite. This is a secondary data analysis so data collection is carried out by a third party, uninvolved in this research. Outcomes are assessed at baseline, at three months and annually thereafter.

Predictors

For peer review only

Candidate predictors to be considered in the prediction models will be based on, the most promising predictor variables as identified in previous systematic reviews in this field (Fahey et al 2016 , Pendlebury and Rothwell 2009), ease and reliability of measurement in clinical setting and theoretical association with the progression of outcomes. The number of variables considered to ensure adequate power based on our target sample size (see below), to avoid overfitting, and to encourage parsimony and applicability of the model in clinical practice. Number, type, definition, method for measurement and handling of candidate predictors in the modelling are listed in the data supplement. All predictors are measured at patient presentation. Data collection is carried out by a third party, not involved in this research.

Sample size

Cumulative survival up to 10 years after stroke is estimated at 63.7%, 42.8% and 24.0%, surviving up to 1, 5 and 10 years respectively (Wolfe et al 2011). Disability is estimated on average at 11% (10 year average and standardised to European population in the SLSR, Wolfe et al 2011). Current SLSR data (1995-2015) estimates disability at 24.6%, 23.7% and 26.3% at 1, 5 and 10 years respectively. Cognitive impairment is estimated at approximately 22% in the first five years follow stroke (age-standardised to European population, Douiri et al 2013). Current SLSR data (1995-2015) estimates cognitive impairment at 29.8%, 28.9%, 28.6% at 1, 5 and 10 years respectively. Rules of thumb for fitting multivariate models suggest that 10 events for every variable (EPV) are required to avoid overfitting in model development studies. Although we are within these limitations, some works have suggested this rule is limited, especially when applied to predictions. Higher EPV may be needed when low-prevalence predictors are present in a model to eliminate bias in regression coefficients and improve predictive accuracy (Ogundimu 2016). Others have highlighted scenarios where the 10 EPV rule can be relaxed. 10:1 rule is a minimum and using a conservative ratio of 20:1 suggested when using penalised regressions (shrinkage of coefficients) (Steyerberg et al 2000), the sample suggested has enough subjects to build a model of recovery with up to 15 predictors. Survival and cognitive impairment models could be explored with even more than 15 variables..

Missing data

Missing variable data will inevitably occur, with not all patients providing all variables of interest. In this situation, multiple imputation will be used to impute, under a missing at random assumption, missing values so to avoid excluding patients from the analysis. Multiple imputation is advocated as the preferred imputation method, and also leads to more correct standard errors and P values; in single imputation, these are estimated too small (low), falsely increasing chance findings (Moons et al 2012, Donders et al 2006, Groenwold et al 2012, Janssen et al 2010, Jansen et al 2009, Moons et al 2006, Sterne et al 2009). However imputations typically break down when missing data is excessive, therefore data with more than 80% missing data will be excluded.

Modelling method

We will develop three clinical prediction models; one for mortality, one for functional outcome and one for cognitive impairment. The start point is time of stroke and end point 5 years post stroke.

For predictors selection during multivariate modelling a variable selection and shrinkage procedure will be used to decide which of the identified candidate predictor variables should be included in the final prediction model. Continuous variables will be kept as continuous (rather than say dichotomising), to avoid a loss of power. Non-linear trends will also be considered using fractional polynomials and the multivariable fractional polynomial procedure. Clinically meaningful interactions will be included in the model.

We will assess internal validity with a bootstrapping procedure for a realistic estimate of the performance of both prediction models in similar future patients (Steyerberg 2009; Harrell et al 1996). Our approach is as follows:

1. Develop the prediction model using the entire original sample and determine the apparent performance.
2. Generate a bootstrap sample, by sampling n individuals with replacement from the original sample.
3. Develop a model using the bootstrap sample (applying all the same modelling and predictor selection methods, as in step 1):
    a. Determine the performance (described below) of this model on the bootstrap sample (bootstrap performance).

For peer review only

   b. Determine the performance of the bootstrap model in the original sample (test performance).
4. Calculate the optimism as the difference between the bootstrap performance and the test performance.
5. Repeat steps 2 through 4 at least 500 times.
6. Average the estimates of optimism in step 5, and subtract the value from the performance obtained in step 1 to obtain an optimism-corrected estimate of performance.

The bootstrap validation approach uses all of the data to develop the prediction model and provides a mechanism to account for model overfitting or uncertainty in the entire model development process, thereby quantifying any optimism in the final prediction model. Also, it provides for estimating a shrinkage factor that can be used to adjust the regression coefficients and apparent performance for optimism, such that better performance will be obtained in subsequent model validation studies and applications (Collins et al 2015). Once a final model is identified, methods will be applied to simplify and adapt the presentation of the model to a scoring system to facilitate its application in practice.

Model performance

Model performance will be assessed in both the derivation and validation datasets. For model development studies, we are primarily interested in discrimination, because the model will be well calibrated (on average) by definition and even perfect when the smoothed method is used. In validation studies, assessment of both discrimination and calibration is fundamental (Steyerberg et al 2010, Vickers and Cronin 2010).

Calibration of the derived models will be measured using calibration plot and Hosmer-Lemeshow test or counterpart test for the survival model (D'Agostino and Nam 2004; Grønnesby and Borgan 1994). Such tests have limited statistical power to evaluate poor calibration and are sensitive to the grouping and sample size (Bertolini et al 2000; Kramer and Zimmerman 2007; Marcin and Romano 2007): they are often nonsignificant for small samples and nearly always significant for large samples. Furthermore, they convey no indication of magnitude or direction of any miscalibration, hence calibration plots will also

For peer review only

be presented. Calibration plots will also be evaluated in relation to key predictors/subgroups.

Discrimination of the derived models will be measured using the concordance statistic (c-statistic) (measures with confidence intervals) (Cook 2008). The c-index is identical to the area under the receiver-operating characteristic curve (AUROC) for models with binary endpoints, and can be generalized for time-to-event (survival) models accounting for censoring. A benchmark level of discrimination was determined in our meta-analysis for mortality and functional outcome. Existing clinical prediction models for post stroke cognitive impairment (n=2) report accuracies ≤0.7. On the basis of the reference standard values in statistics literature (Hosmer 2004) and those of previous work in stroke, we will consider AUC values of greater than 0.8 to be acceptable for these outcomes. Classification measures (e.g., sensitivity, specificity, predictive values, net reclassification improvement) will be presented and cut points selected a-priori. To assess clinical utility Decision curve Analysis will also be undertaken (Van Calster and Vickers 2014; Vickers and Elkin 2006;Baker 2009, Baker et al 2009, Baker and Kramer 2012).

Model Evaluation

Developed models will be validated temporally using the SLSR. To provide evidence evidence that the model performs well for other (external) patients we will externally validate in data collected by the Dijon Stroke Register(DSR). This geographical validation will enable us to assess the prognostic performance and the generalisability of the model. The methods of the DSR have been described previously in detail (Giroud 1997; Bejot 2013; Lainay et al 2015) and both datasets are contrasted here. The Dijon Stroke Registry is also a population-based registry, conducted among the residents of the city of Dijon, France (2007 census: 151 543 inhabitants), since 1985. Stroke is defined according to WHO diagnostic criteria in both the DSR and the SLSR.

We will update the model if it shows poor performance to adjust to the new situation by recalibration or revision methods depending on discrimination performance (c-statistics <0.80). If model is updated updating approaches recommended by Steyerberg (model recalibration, model revision, model extension) will be adopted as appropriate (Steyerberg 2013).

For peer review only

CONCLUSION

We have described the methods and statistical analysis plan to develop and validate a family of clinical prediction models for stroke over the long term. To our knowledge, this tool will be the first of its kind in stroke to follow, a priori, the PROGRESS framework and TRIPOD reporting guidelines for prognostic research. Importantly for secondary prevention, the tool will be developed specifically to predict the progression of disease and identify those at high risk of an adverse outcome. . Results coming from this study will be interpreted for both clinical and research purposes.

Funding

We acknowledge financial support from the Division of Health and Social Care Research at King's College London, the National Institute for Health Research (NIHR) Biomedical Research and from the NIHR Collaboration for Leadership in Applied Health Research and Care South London at King's College Hospital NHS Foundation Trust

Contributorship statement

MF contributed to design and planning of the study, writing and reviewing the manuscript. AD and CW Supervised development of work, evaluated and edited the manuscript. AR and YB evaluated and edited the manuscript.

Competing interests

None declared.

Data sharing statement

No additional data are available.

For peer review only

REFERENCES

Altman DG, Vergouwe Y, Royston P, et al. Prognosis and prognostic research: validating a prognostic model. BMJ 2009;338:b605.

Baker SG. Putting risk prediction in perspective: relative utility curves. J Natl Cancer Inst. 2009;101:1538-42.

Baker SG, Cook NR, Vickers A, Kramer BS. Using relative utility curves to evaluate risk prediction. J R Stat Soc Ser A Stat Soc. 2009; 172:729-48.

Baker SG, Kramer BS. Evaluating a new marker for risk prediction: decision analysis to the rescue. Discov Med. 2012;14:181-8.

Bejot, Y., Daubail, B., Jacquin, A., Durier, J., Osseby, G., Rouaud, O. and Giroud, M. (2013). Trends in the incidence of ischaemic stroke in young adults between 1985 and 2011: the Dijon Stroke Registry. Journal of Neurology, Neurosurgery & Psychiatry, 85(5), pp.509-513.

Bouwmeester W, Zuithoff NP, Mallett S, et al. Reporting and methods in clinical prediction research: a systematic review. PLoS Med2012;9:e1001221. doi: 10.1371/journal.pmed.1001221 pmid:22629234.

Bertolini G, D'Amico R, Nardi D, Tinazzi A, Apolone G. One model, several results: the paradox of the Hosmer-Lemeshow goodness-of-fit test for the logistic regression model. J Epidemiol Biostat. 2000;5:251-3.

Collins, G., Reitsma, J., Altman, D. and Moons, K. (2015). Transparent Reporting of a Multivariable Prediction Model for Individual Prognosis or Diagnosis (TRIPOD): The TRIPOD Statement. European Urology, 67(6), pp.1142-1151.

Collins GS, Altman DG. Identifying patients with undetected renal tract cancer in primary care: an independent and external validation of QCancer® (Renal) prediction model. Cancer Epidemiol. 2013; 37:115-20.

Cook NR. Statistical evaluation of prognostic versus diagnostic models: beyond the ROC curve. Clin Chem. 2008;54:17-23.

For peer review only

Counsell, C., & Dennis, M. (2001). Systematic review of Prognostic models in patients with acute stroke. Cerebrovascular Diseases, 12(3), 159–170. doi:10.1159/000047699

D'Agostino RB, Nam BH. Evaluation of the performance of survival analysis models: discrimination and calibration measures. In: Balakrishnan N, Rao CR, eds. Handbook of Statistics, Survival Methods. Amsterdam: Elsevier; 2004:1-25.

Donders AR, van der Heijden GJ, Stijnen T, Moons KG. Review: a gentle introduction to imputation of missing values. J Clin Epidemiol. 2006;59:1087-91.

Dorresteijn JA, Visseren FL, Ridker PM, Wassink AM, Paynter NP, Steyerberg EW, et al. Estimating treatment effects for individual patients based on the results of randomised clinical trials. BMJ. 2011; 343:d5888

Groenwold RH, White IR, Donders AR, Carpenter JR, Altman DG, Moons KG. Missing covariate data in clinical research: when and when not to use the missing-indicator method for analysis. CMAJ. 2012;184:1265-9.

Grønnesby JK, Borgan O. A method for checking regression models in survival analysis based on the risk score. Lifetime Data Anal. 1996;2:315-28.

Fahey, M., Crayton, E Charles, W. and Abdel, D. (2016). Clinical Prediction Models for Ischemic Stroke Recovery: A Systematic Review and Meta-Analysis. European Stroke Journal, 1(1 Suppl), pp.3-612.

Feigin, V.L., Forouzanfar, M.H., Krishnamurthi, R., Mensah, G.A., Connor, M., Bennett, D.A., Moran, A.E., Sacco, R.L., Anderson, L., Truelsen, T., O'Donnell, M., Venketasubramanian, N., Barker-Collo, S., Lawes, C.M.M., Wang, W., Shinohara, Y., Witt, E., Ezzati, M., Naghavi, M. and Murray, C. (2014) 'Global and regional burden of stroke during 1990–2010: Findings from the global burden of disease study 2010', The Lancet, 383(9913), pp. 245–255. doi: 10.1016/s0140-6736(13)61953-4.

Giroud, M., Lemesle, M., Quantin, C., Vourch, M., Becker, F., Milan, C., Brunet-Lecomte, P. and Dumas, R. (1996). A Hospital-Based and a Population-Based Stroke Registry Yield Different Results: The Experience in Dijon, France. Neuroepidemiology, 16(1), pp.15-21.

For peer review only

Hayward RA, Kent DM, Vijan S, Hofer TP. Multivariable risk prediction can greatly enhance the statistical power of clinical trial subgroup analysis. BMC Med Res Methodol. 2006;6:18.

Harrell FE Jr, Lee KL, Mark DB. Multivariable prognostic models: issues in developing models, evaluating assumptions and adequacy, and measuring and reducing errors. Stat Med. 1996;15:361-87.

Hosmer, D. and Lemeshow, S. (1989). Applied logistic regression. New York: Wiley.

Janssen KJ, Donders AR, Harrell FE, Vergouwe Y, Chen Q, Grobbee DE, et al. Missing covariate data in medical research: to impute is better than to ignore. J Clin Epidemiol. 2010;63:721-7

Janssen KJ, Vergouwe Y, Donders AR, Harrell FE, Chen Q, Grobbee DE, et al. Dealing with missing predictor values when applying clinical prediction models. Clin Chem. 2009;55:994-1001.

Kamper SJ, Hancock MJ, Maher CG. Optimal designs for prediction studies of whiplash. Spine (Phila Pa 1976) 2011;36(25 Suppl):S268–74. doi:10.1097/BRS.0b013e3182388202

Kattan MW, Vickers AJ. Incorporating predictions of individual patient risk in clinical trials. Urol Oncol. 2004;22:348-52.

Kent DM, Hayward RA. Limitations of applying summary results of clinical trials to individual patients: the need for risk stratification. JAMA. 2007;298:1209-12.

Kwakkel, G., Wagenaar, R.C., Kollen, B.J. and Lankhorst, G.J., 1996. Predicting disability in stroke—a critical review of the literature. Age and ageing, 25(6), pp.479-489.

Kramer AA, Zimmerman JE. Assessing the calibration of mortality benchmarks in critical care: the Hosmer-Lemeshow test revisited. Crit Care Med. 2007;35:2052-6.

Langhorne, P., Bernhardt, J. and Kwakkel, G., 2011. Stroke rehabilitation. The Lancet, 377(9778), pp.1693-1702.

Lainay, C., Benzenine, E., Durier, J., Daubail, B., Giroud, M., Quantin, C. and Bejot, Y. (2014). Hospitalization Within the First Year After Stroke: The Dijon Stroke Registry. Stroke, 46(1), pp.190-196.

For peer review only

Moons KG, Kengne AP, Woodward M, Royston P, Vergouwe Y, Altman DG, et al. Risk prediction models: I. Development, internal validation, and assessing the incremental value of a new (bio)marker. Heart. 2012;98:683-90.

Moons, K., de Groot, J., Bouwmeester, W., Vergouwe, Y., Mallett, S., Altman, D., Reitsma, J. and Collins, G. (2014). Critical Appraisal and Data Extraction for Systematic Reviews of Prediction Modelling Studies: The CHARMS Checklist. PLoS Med, 11(10), p.e1001744.Moons

Moons KG, Donders RA, Stijnen T, Harrell FE Jr. Using the outcome for imputation of missing predictor values was preferred. J Clin Epidemiol. 2006;59:1092-101.

Marcin JP, Romano PS. Size matters to a model's fit. Crit Care Med. 2007;35:2212-3.

KG, Royston P, Vergouwe Y, Grobbee DE, Altman DG. Prognosis and prognostic research: what, why, and how? BMJ. 2009; 338:b375.

Ogundimu, E., Altman, D. and Collins, G. (2016). Adequate sample size for developing prediction models is not simply related to events per variable. Journal of Clinical Epidemiology, 76, pp.175-182.

Pendlebury ST, Rothwell PM. Prevalence, incidence, and factors associated with pre-stroke and post-stroke dementia: a systematic review and meta-analysis. Lancet Neurol. 2009;8:1006–1018.

Riley RD, Hayden JA, Steyerberg EW, Moons KG, Abrams K, Kyzas PA, et al; PROGRESS Group. Prognosis Research Strategy (PROGRESS) 2: prognostic factor research. PLoS Med. 2013;10:

e1001380.

Royston P, Moons KGM, Altman DG, Vergouwe Y. Prognosis and prognostic research: Developing a prognostic model. BMJ2009;338:b604. doi:10.1136/bmj.b604 pmid:19336487.

Steyerberg, E. W.; Eijkemans, M. J.; Harrell, F. E. Jr.; Habbema, J. D. (2000). "Prognostic modelling with logistic regression analysis: a comparison of selection and estimation methods in small data sets". Stat Med. 19: 1059–1079. doi:10.1002/(sici)1097-0258(20000430)19:8<1059::aid-sim412>3.0.co;2-0.

For peer review only

Steyerberg, E. (2009). Clinical prediction models A Practical Approach to Development, Validation, and Updating. New York: Springer.

Steyerberg EW, Moons KGM, van der Windt DA, et al. Prognosis Research Strategy (PROGRESS) 3: prognostic model research. PLoS Med 2013;10:e1001381.

Steyerberg EW, Vickers AJ, Cook NR, Gerds T, Gonen M, Obuchowski N, et al. Assessing the performance of prediction models: a framework for traditional and novel measures. Epidemiology. 2010; 21:128-38.

Sterne JA, White IR, Carlin JB, Spratt M, Royston P, Kenward MG, et al. Multiple imputation for missing data in epidemiological and clinical research: potential and pitfalls. BMJ. 2009;338:b2393.

Van Calster B, Vickers AJ. Calibration of risk prediction models: impact on decision-analytic performance. Med Decis Making. 2014 Aug 25.

Veerbeek, J.M., Kwakkel, G., van Wegen, E.E., Ket, J.C. and Heymans, M.W., 2011. Early prediction of outcome of activities of daily living after stroke a systematic review. Stroke, 42(5), pp.1482-1488.

Vickers AJ, Cronin AM. Everything you always wanted to know about evaluating prediction models (but were too afraid to ask). Urology. 2010;76:1298-301.

Vickers AJ, Elkin EB. Decision curve analysis: a novel method for evaluating prediction models. Med Decis Making. 2006;26:565-74.

Wasson JH, Sox HC, Neff RK, Goldman L. Clinical prediction rules. Applications and methodological standards. N Engl J Med. 1985; 313:793-9.

Winstein, C.J., Stein, J., Arena, R., Bates, B., Cherney, L.R., Cramer, S.C., Deruyter, F., Eng, J.J., Fisher, B., Harvey, R.L. and Lang, C.E., 2016. Guidelines for Adult Stroke Rehabilitation and Recovery A Guideline for Healthcare Professionals From the American Heart Association/American Stroke Association. Stroke, 47(6), pp.e98-e169.

For peer review only

DEVELOPMENT AND VALIDATION OF CLINICAL PREDICTION MODELS FOR MORTALITY, FUNCTIONAL OUTCOME AND COGNITIVE IMPAIRMENT AFTER STROKE: A STUDY PROTOCOL

Journal:	*BMJ Open*
Manuscript ID	bmjopen-2016-014607.R1
Article Type:	Protocol
Date Submitted by the Author:	08-Feb-2017
Complete List of Authors:	Fahey, Marion; King's College London, Division of Health and Social Care Research ;<br>Rudd, Anthony; King's College London<br>Béjot, Yannick; Dijon Stroke Registry, EA4184, University Hospital and Medical School of Dijon, neurology<br>Wolfe, Charles; Kings College London, Division of Health and Social Care Research<br>Douiri, Abdel; King's College London, Division of Health and Social Care Research
**Primary Subject Heading**:	Cardiovascular medicine
Secondary Subject Heading:	Epidemiology, Cardiovascular medicine, Patient-centred medicine, Public health
Keywords:	prognosis, Clinical prediction model, Stroke < NEUROLOGY, stratified medicine

SCHOLARONE™ Manuscripts

For peer review only

For peer review only - http://bmjopen.bmj.com/site/about/guidelines.xhtml

DEVELOPMENT AND VALIDATION OF CLINICAL PREDICTION MODELS FOR MORTALITY, FUNCTIONAL OUTCOME AND COGNITIVE IMPAIRMENT AFTER STROKE: A STUDY PROTOCOL

Fahey M[1], Rudd AG[1,3], Bejot Y[4], Wolfe C.D.A [1,2], Douiri A[1,2],

Division of Health and Social Care, King's College London, London

[1]Division of Health and Social Care Research King's College London, London, UK

[2]National Institute for Health Research (NIHR) Collaboration for Leadership in Applied Health Research and Care (CLAHRC) South London at King's College Hospital NHS Foundation Trust, UK

[3]National Institute for Health Research (NIHR) Biomedical Research Centre, Guy's and St Thomas' NHS Foundation Trust, London, UK

[4]Registre Dijonnais des AVC, EA4184, Dijon, France.

Corresponding author: Marion Fahey

Corresponding author's address: Division of Health and Social Care Research, Addison House, Guy's Campus, King's College London, London SE1 1UL, UK

Corresponding author's phone and fax: Phone: +44 20 7848 8732;

Corresponding author's e-mail address: marion.fahey@kcl.ac.uk

Word count, excluding title page, abstract, references, figures and tables: 2,845

For peer review only

Abstract

Introduction:

Stroke is a leading cause of adult disability and death worldwide. The neurological impairments associated with stroke prevent patients from performing basic daily activities and have enormous impact on families and caregivers. Practical and accurate tools to assist in predicting outcome after stroke at patient level can provide significant aid for patient management. Furthermore, prediction models of this kind can be useful for clinical research, health economics, policy making, and clinical decision support.

Methods:

2,869 patients with first ever stroke from South London Stroke Register (SLSR) (1995-2004) will be included in the development cohort. We will use information captured after baseline to construct multilevel models and a Cox proportional hazard model to predict cognitive impairment, functional outcome and mortality up to 5 years after stroke. Repeated random sub-sampling validation (Monte Carlo cross-validation) will be evaluated in model development. Data from participants recruited to the stroke register (2005-2014) will be used for temporal validation of the models. Data from participants recruited to the Dijon Stroke Register (1985-2015) will be used for external validation. Discrimination, calibration and clinical utility of the models will be presented.

Ethics:

Patients, or for patients who cannot consent their relatives, gave written informed consent to participate in stroke-related studies within the SLSR. The SLSR design was approved by the ethics committees of Guy's and St Thomas' NHS Foundation Trust, Kings College

For peer review only - http://bmjopen.bmj.com/site/about/guidelines.xhtml

Hospital, Queens Square, and Westminster Hospitals (London). The Dijon Stroke Registry was approved by the Comité National des Registres and the InVS and has authorization of the Commission Nationale de l'Informatique et des Libertés.

For peer review only

For peer review only - http://bmjopen.bmj.com/site/about/guidelines.xhtml

SUMMARY

Strengths and limitations of this study

- First prognostic tool in Stroke to follow, a priori, the Prognosis Research Strategy (PROGRESS) framework, and Transparent Reporting of a multivariable prediction model for Individual Prognosis or Diagnosis (TRIPOD) reporting guidelines for prognostic research.
- Specifies statistical analysis plan and informative levels of tool performance to increase transparency of results and the final report.
- The proposed study predicts outcomes longitudinally, which may be more reflective of clinical needs than predictions made at pre-defined time points.
- The proposed study is restricted by the use of predictor variables measured in previous data sets and the limitations of these measures. The external validation is not independent.

For peer review only

INTRODUCTION

Stroke is among the most common causes of serious adult physical disability and the third most common cause of death worldwide.[1] Despite the introduction of effective treatments for acute stroke, early rehabilitation and secondary prevention, the majority of stroke survivors have medical comorbidities, physical and/or cognitive impairments that require ongoing active assessment and management.[2] Stroke can be seen as a chronic condition, spanning not only the incident event and formal rehabilitation but the rest of the patient’s life. Rehabilitation from stroke requires a sustained, co-ordinated effort from informed multidisciplinary Teams (MDTs) as well as patients and carers, both in the clinical setting and in the community.[3] MDTs and patients make numerous decisions on the basis of an estimated probability that a specific event will occur in the future. These predictions are used for planning lifestyle or therapeutic decisions on the basis of the risk of developing a particular outcome or state of health.[4-6] More recently such estimates are used to risk-stratify participants in therapeutic intervention trials and case mix classifications.[7,8]

Information from a single predictor is often insufficient to provide reliable estimates of prognostic probabilities or risks, particularly in complex patients, for example those with comorbidities.[9,10] Therefore probability estimates are commonly based on combining information from multiple predictors to form a multivariable clinical prediction model (CPM).[11,12]

CPMs are abundant in prognostic research literature but few are implemented or used in routine clinical practice.[13] One explanation for this is that, although many models have been developed,[14,15]they have limited utility for clinical applications, particularly in a long term care setting. Progression or regression of disease is highly variable both over time and

For peer review only - http://bmjopen.bmj.com/site/about/guidelines.xhtml

between individuals.[16] Current clinical prediction models typically estimate risk at predefined time points. With variability in mind, time series data and methods may be more appropriate for accurately capturing recovery, particularly when the aim is planning immediate and long term care simultaneously for individual patients.

There is generally a lack of confidence among clinicians in applying risk scores in practice. Many believe there is the lack of sufficient evidence to demonstrate the reproducibility and transportability of the model in a different population (Altman 2009). To be considered useful, a risk score should be clinically credible, accurate (well calibrated with good discriminative ability) and have generality (be externally validated).[17]

We have evaluated the accuracy of existing models in predicting stroke outcomes by systematic review of literature and meta-analysis. The systematic review and meta analysis concluded that existing models have much potential and advised to build on previous work, opposed to designing new models, in developing clinical prediction models suitable for the long term care setting.[15] Models predicting outcomes longitudinally, opposed to pre-defined time points, may best support patient management and so we will build on work by colleagues in this area.[18,19]

For peer review only

OBJECTIVES

Primary objectives

To develop and to internally validate prediction models in patients with ischemic stroke for functional outcome, survival and cognitive impairment at five years.

For peer review only - http://bmjopen.bmj.com/site/about/guidelines.xhtml

To externally validate and to update these models through an external dataset of ischemic stroke patients.

Secondary objectives

To Assess deviations of predicted recovery curves.

To investigate why stroke patients regain different recovery that plateaued at different levels.

To derive and validate scoring system for classifying risks (case-mix classification) from these patient-centred predictive models

METHODS

This cohort study is informed by the PROGRESS framework and recommendations by authors in the field. We will report the study protocol using the Transparent Reporting of a multivariable prediction model for Individual Prognosis Or Diagnosis (TRIPOD) statement for prediction studies.[20]

Source of data

The study is a prospective longitudinal cohort study. The data source is the South London Stroke Register(SLSR) an ongoing, prospective population-based stroke register set up in January 1995 recording all first-ever strokes in patients of all ages for an inner area of South London. The methods of the SLSR have been described previously in detail[2]and are summarized here. Stroke was defined according to the World Health Organisation criteria and Oxfordshire Community Stroke Project Classification. Multiple over-lapping sources of information are employed. Data from 1995 to 2016 will be used for model derivation and internal validation.

For peer review only - http://bmjopen.bmj.com/site/about/guidelines.xhtml

Participants

Patients admitted to hospitals serving the study area (two teaching hospitals within and three hospitals outside the study area) are identified by regular reviews of acute wards admitting stroke patients, national data on patients admitted to any hospital in England and Wales with a diagnosis of stroke are screened for additional patients. All general practitioners (N=699 (2011)) within and on the borders of the study area are contacted regularly and asked to notify the SLSR of stroke patients. Referral of non-hospitalised stroke patients to a neurovascular outpatient clinic (from 2003) or domiciliary visit to patients by the study team is also available to general practitioners. Community therapists are contacted every 3 months. Death certificates are checked regularly. Patients are assessed at the stroke onset, 3 months and annually after stroke. Participants receive treatments in line with UK national clinical guidelines for stroke. Completeness of case ascertainment has been estimated at 88% by a multinomial-logit capture-recapture model using the methods described in detail elsewhere.[21]

Outcome

The outcomes of interest are Mortality measured as time to event, functional outcome measured using the Bartel Index and Cognitive Impairment measured using the Mini Mental State Exam or Abbreviated Mental Test measured at baseline, 3 months and annually up to 5 years after stroke. Specially trained nurses and field workers collect all data prospectively whenever feasible. A study doctor verifies the diagnosis of stroke. Patients are examined within 48 hours of referral to the SLSR where possible. Follow up data are collected by validated postal or face-to-face instruments with patients and/or their carers. Outcome

For peer review only - http://bmjopen.bmj.com/site/about/guidelines.xhtml

definition and measurement method is the same for all patients. Data collection is carried out by a third party, uninvolved in this study.

Predictors

Candidate predictors to be considered in the prediction models will be based on predictor variables as identified in previous systematic reviews in this field,[15,22] ease and reliability of measurement in clinical setting and theoretical association with the progression of outcomes. The number of variables required to ensure adequate power based on our target sample size (see below), to avoid overfitting, and to encourage parsimony and applicability of the model in clinical practice will also be considered. Number, type, definition, method for measurement and handling of candidate predictors in the modelling are listed in the data supplement. All predictors are measured at patient presentation. Data collection is carried out by a third party, not involved in this research.

Sample size

Cumulative survival up to 10 years after stroke is estimated at 63.7%, 42.8% and 24.0%, surviving up to 1, 5 and 10 years respectively.[2] Disability is estimated on average at 11% (10 year average and standardised to European population in the SLSR).[2] Current SLSR data (1995-2015) estimates disability (Bartel Index > 15) at 24.6%, 23.7% and 26.3% at 1, 5 and 10 years respectively. Cognitive impairment (Mini mental State Exam <24) is estimated at approximately 22% in the first five years follow stroke (age-standardised to European population).[23] Current SLSR data (1995-2015) estimates cognitive impairment at 29.8%, 28.9%, 28.6% at 1, 5 and 10 years respectively. Rules of thumb for fitting multivariate models suggest that 10 events for every variable (EPV) are required to avoid overfitting in

For peer review only - http://bmjopen.bmj.com/site/about/guidelines.xhtml

model development studies. Although we are within these limitations, the use of this rule can result in small sample sizes which may lead to overfitting and optimism. It is recommended that EPV should be data driven.[24] Sample size calculations described by Jinks will be used for survival analysis.[25] Simulation will be used to determine appropriate sample size for models prediction functional outcome and cognitive impairment.

Missing data

Missing data is inevitable in studies with long follow up and may lead to bias and imprecision. Multiple imputation will be used to impute missing values, under a missing at random assumption, so as to reduce bias and avoid excluding participants from the analysis.[26-30] Imputations typically break down when missing data is excessive, therefore data with more than 80% missing data will be excluded.

Statistical analysis methods

We will develop three clinical prediction models for the outcomes : (i) mortality , (ii) functional outcome and (iii) cognitive impairment up to five years after stroke. The start point is time of stroke and end point 5 years post stroke.

For predictor selection during multivariate modelling a variable selection and shrinkage procedure will be used to decide which of the identified candidate predictor variables should be included in the final prediction model. Continuous variables will be kept as continuous (rather than say dichotomising), to avoid a loss of power. Non-linear trends will be considered using fractional polynomials and the multivariable fractional polynomial procedure. Clinically meaningful interactions (e.g time) will be included in the model.

For peer review only - http://bmjopen.bmj.com/site/about/guidelines.xhtml

For peer review only

We will assess internal validity with a bootstrapping procedure for a realistic estimate of the performance of prediction models in similar future patients.[5,12] The bootstrap validation approach uses all of the data to develop the prediction model and provides a mechanism to account for model overfitting or uncertainty in the entire model development process, thereby quantifying any optimism in the final prediction model. Also, it provides for estimating a shrinkage factor that can be used to adjust the regression coefficients and apparent performance for optimism, such that better performance will be obtained in subsequent model validation studies and applications.[31]

External validity will be assessed using data form the Dijon Stroke Register (DSR). Patients will be classified using the estimates of the previously developed models and performance assessed. Subgroup analyses will be carried out to assess deviations from predicted recovery trajectories and investigate why stroke patients regain different recovery that plateaued at different levels.

Risk groups

Although risk groups (e.g. “high risk”, “moderate risk”, “low risk”) may make models more accessible, no risk groups will be created. There is no clear consensus on how to create risk groups or how many groups to use.[31] There are concerns that use of risk groups may not be in the best interest of patients and may become standard although lacking any rational.[31] Also, the simplification of predicted probabilities assumes risk is the same for all individuals within that category.

Development vs. Validation

For peer review only - http://bmjopen.bmj.com/site/about/guidelines.xhtml

Data from the Dijon Stroke Register (DSR) will be used for external validation; it's methods have been described previously in detail[32-34] and both datasets are contrasted here. The DSR and SLSR are population-based registers, multiple overlapping sources of notification are used and stroke is defined according to World Health organisation criteria for both. Specially trained field workers collect all data. This includes sociodemographic factors, disease characteristics patient history and cardiovascular risk factors in both registers. DSR participants are followed up at time of stroke and yearly thereafter; these questionnaires are administered in out-patient clinics or conducted telephonically by clinical nurses. Follow up procedures in SLSR are similar but participants are followed up at three months also. In both registers, survival is measured using national data, Cognitive status is measured using either the Abbreviated Mental Test (AMT) or the Mini Mental State Exam (MMSE) and disability is measured using Barthel Index (BI).

REPORTING OF RESULTS

Participants

The flow of participants through the study, including the number of participants with and without the outcome and a summary of the follow-up time will be described. The characteristics of the participants including the number of participants with missing data for predictors and outcome for both development and validation cohorts will be provided.

Model development

The number of participants and outcome events in each analysis will be presented as well as the unadjusted association between each candidate predictor and outcome.

For peer review only - http://bmjopen.bmj.com/site/about/guidelines.xhtml

Model specification

The full prediction model for each outcome will be presented including all regression coefficients, and model intercept and baseline survival. Once a final model is identified, methods will be applied to simplify and adapt the presentation of the model to a scoring system to facilitate its application in practice at a later date. An explanation of how to use the model and scoring system will also be presented.

Model performance

Model performance assessment has been designed using the framework described by Steyerberg and colleagues.[35] Model performance will be assessed in derivation and validation datasets. For model development studies, we are primarily interested in discrimination, because the model will be well calibrated (on average) by definition when smoothing methods are used. In validation studies, assessment of both discrimination and calibration is fundamental.[35,36] Calibration of the derived models will be measured using calibration plot and Hosmer-Lemeshow test or counterpart test for the survival model.[37,38] Furthermore, they convey no indication of magnitude or direction of any miscalibration, hence calibration plots will also be presented. Calibration plots will also be evaluated in relation to key predictors/subgroups. Discrimination of the derived models will be measured using the concordance statistic and confidence intervals (c-statistic). The c-index is identical to the area under the receiver-operating characteristic curve (AUROC) for models with binary endpoints, and can be generalized for time-to-event (survival) models accounting for censoring. A benchmark level of discrimination was determined in our meta-analysis for mortality and functional outcome. On the basis of the reference standard values

For peer review only - http://bmjopen.bmj.com/site/about/guidelines.xhtml

in statistics literature[39] and those of previous work in stroke, we will consider AUC values of greater than 0.8 to be acceptable for these outcomes. Classification measures (e.g., sensitivity, specificity, predictive values, net reclassification improvement) will be presented and cut points selected a-priori. Decision curve Analysis will be undertaken to assess clinical utility.[40]

Model updating

We will update the model if it shows poor performance in external data(DSR) by recalibration or revision methods depending on discrimination performance (c-statistics <0.80). If model is updated updating approaches recommended by Steyerberg (model recalibration, model revision, model extension) will be adopted as appropriate.[13]

CONCLUSION

We have described the methods and statistical analysis plan to develop and to validate a family of clinical prediction models for stroke over the long term. To our knowledge, this tool will be the first of its kind in stroke to follow, a priori, the PROGRESS framework and TRIPOD reporting guidelines for prognostic research. Importantly for secondary prevention, the tool will be developed specifically to predict the progression of disease and to identify those at high risk of an adverse outcome. Results coming from this study will be interpreted for both clinical and research purposes.

FUNDING

We acknowledge financial support from the Division of Health and Social Care Research at King's College London, the National Institute for Health Research (NIHR) Biomedical

For peer review only - http://bmjopen.bmj.com/site/about/guidelines.xhtml

Research and from the NIHR Collaboration for Leadership in Applied Health Research and Care South London at King's College Hospital NHS Foundation Trust

CONTRIBUTORSHIP STATEMENT

MF contributed to design and planning of the study, writing and reviewing the manuscript. AD and CW supervised development of work, evaluated and edited the manuscript. AR and YB evaluated and edited the manuscript.

COMPETING INTERESTS

None declared.

DATA SHARING STATEMENT

No additional data are available.

For peer review only

For peer review only - http://bmjopen.bmj.com/site/about/guidelines.xhtml

REFERENCES

1. Feigin VL, Forouzanfar MH, Krishnamurthi R et al. Global and regional burden of stroke during 1990-2010: findings from the Global Burden of Disease Study 2010. *Lancet* 2014;383:245-254.

2. Wolfe CD, Crichton SL, Heuschmann PU et al. Estimates of outcomes up to ten years after stroke: analysis from the prospective South London Stroke Register. *PLoS Med* 2011;8:e1001033.

3.Winstein CJ, Stein J, Arena R et al. Guidelines for Adult Stroke Rehabilitation and Recovery: A Guideline for Healthcare Professionals From the American Heart Association/American Stroke Association. *Stroke* 2016;47:e98-e169.

4. Moons KGM, Altman DG, Vergouwe Y et al. Prognosis and prognostic research: application and impact of prognostic models in clinical practice. *BMJ* 2009;338: b606

5. Steyerberg EW. Clinical Prediction Models: A Practical Approach to Development, Validation, and Updating. New York Springer 2009.

6. Wasson JH, Sox HC, Neff RK et al. Clinical prediction rules. Applications and methodological standards. *N Engl J Med* 1985;313:793-799.

7. Dorresteijn JA, Visseren FL, Ridker PM et al. Estimating treatment effects for individual patients based on the results of randomised clinical trials. *BMJ* 2011;343:d5888.

8. Kattan MW, Vickers AJ. Incorporating predictions of individual patient risk in clinical trials. *Urol Oncol* 2004;22:348-352.

For peer review only

For peer review only - http://bmjopen.bmj.com/site/about/guidelines.xhtml

9. Riley RD, Hayden JA, Steyerberg EW et al. Prognosis Research Strategy (PROGRESS) 2: prognostic factor research. *PLoS Med* 2013;10:e1001380.

10. Collins GS, Omar O, Shanyinde M et al. A systematic review finds prediction models for chronic kidney were poorly reported and often developed using inappropriate methods. *J Clin Epidemiol* 2013;268-277.

11. Royston P, Altman DG. External validation of a cox prognostic model: principles and methods. *BMC Med Res Methodol* 2013;13:33.

12. Harrell FE, Lee KL, Mark DB. Multivariable prognostic models: issues in developing models, evaluating assumptions and adequacy, and measuring and reducing errors. *Stat Med* 1996;15:361-387.

13. Steyerberg EW, Moons KG, van der Windt DA et al. Prognosis Research Strategy (PROGRESS) 3: prognostic model research. *PLoS Med* 2013;10:e1001381.

14. Counsell C, Dennis M. Systematic review of prognostic models in patients with acute stroke. *Cerebrovasc Dis* 2001;12:159-170.

15. Fahey M, Crayton E, Charles W et al. Oral Abstracts: Clinical Prediction Models for Ischemic Stroke Recovery: A Systematic Review and Meta-Analysis. *European Stroke Journal* 2016;1:613-780.

16. Langhorne P, Bernhardt J, Kwakkel G. Stroke rehabilitation. *Lancet* 2011;377:1693-1702.

17. Altman DG. Prognostic models: a methodological framework and review of models for breast cancer. *Cancer Invest* 2009;27:235-243.

For peer review only

18. Tilling K, Sterne JA, Wolfe CD. Multilevel growth curve models with covariate effects: application to recovery after stroke. *Stat Med.* 2001;20:685-704.

19. Tilling K, Sterne JA, Rudd AG et al. A new method for predicting recovery after stroke. *Stroke* 2001;32:2867-73.

20. Moons KG, Altman DG, Reitsma JB et al. New Guideline for the Reporting of Studies Developing, Validating, or Updating a Multivariable Clinical Prediction Model: The TRIPOD Statement. *Adv Anat Pathol* 2015;22:303-305.

21. Tilling K, Sterne JA, Wolfe CD. Estimation of the incidence of stroke using a capture-recapture model including covariates. *Int J Epidemiol* 2001;30:1351–1359.

22. Pendlebury ST, Rothwell PM. Prevalence, incidence, and factors associated with pre-stroke and post-stroke dementia: a systematic review and meta-analysis. *Lancet Neurol* 2009;8:1006-1018.

23. Douiri A, Rudd AG, Wolfe CD. Prevalence of post stroke cognitive impairment: South London Stroke Register 1995-2010. *Stroke* 2013;44:138-145.

24. Ogundimu EO, Altman DG, Collins GS. Adequate sample size for developing prediction models is not simply related to events per variable. *J Clin Epidemiol* 2016;76:175-182.

25. Jinks R. Sample size for multivariable prognostic models [Doctoral Thesis]. University College London; 2012.

26. Donders AR, van der Heijden GJ, Stijnen T et al. Review: a gentle introduction to imputation of missing values. *J Clin Epidemiol* 2006;59:1087-1091.

For peer review only

For peer review only - http://bmjopen.bmj.com/site/about/guidelines.xhtml

27. Groenwold RH, White IR, Donders AR et al. Missing covariate data in clinical research: when and when not to use the missing-indicator method for analysis. *CMAJ* 2012;184:1265-1269.

28. Janssen KJ, Vergouwe Y, Donders AR et al. Dealing with missing predictor values when applying clinical prediction models. *Clin Chem* 2009;55:994-1001.

29. Moons KG, Donders RA, Stijnen T et al. Using the outcome for imputation of missing predictor values was preferred. *J Clin Epidemiol* 2006;59:1092-1101.

30. Sterne JAC, White IR, Carlin JB et al. Multiple imputation for missing data in epidemiological and clinical research: potential and pitfalls. *BMJ* 2009;338;b2393.

31. Moons KG, Altman DG, Reitsma JB et al. Transparent Reporting of a multivariable prediction model for Individual Prognosis Or Diagnosis (TRIPOD): explanation and elaboration. *Ann Intern Med* 2015;163:w1-73.

32. Giroud M, Lemesle M, Quantin C et al. A hospital-based and a population-based stroke registry yield different results: the experience in Dijon, France. *Neuroepidemiology* 1997;16:15-21.

33. Bejot Y, Daubail B, Jacquin A et al. Trends in the incidence of ischaemic stroke in young adults between 1985 and 2011: the Dijon Stroke Registry. *J Neurol Neurosurg Psychiatry* 2014;85:509-513.

34. Lainay C, Benzenine E, Durier J et al. Hospitalization within the first year after stroke: the Dijon stroke registry. *Stroke* 2015;46:190-196.

35. Steyerberg EW, Vickers AJ, Cook NR et al. Assessing the performance of prediction models: a framework for traditional and novel measures. *Epidemiology* 2010;21.

For peer review only - http://bmjopen.bmj.com/site/about/guidelines.xhtml

For peer review only

36.Vickers AJ, Cronin AM. Everything you always wanted to know about evaluating prediction models (but were too afraid to ask). *Urology* 2010;76:1298-1301.

37. D'Agostino RB, Nam B-H. Evaluation of the Performance of Survival Analysis Models: Discrimination and Calibration Measures. Handbook of Statistics 2003;23:1-25.

38. Gronnesby JK, Borgan O. A method for checking regression models in survival analysis based on the risk score. *Lifetime Data Anal* 1996;2:315-328.

39. Hosmer DW, Lemeshow S, Sturdivant R. Applied Logistic Regression. Wiley 2013.

40.Vickers AJ, Elkin EB. Decision curve analysis: a novel method for evaluating prediction models. *Med Decis Making* 2006;26:565-574.

For peer review only

For peer review only - http://bmjopen.bmj.com/site/about/guidelines.xhtml
